# Chromogranin A and the **α** -subunit of glycoprotein hormones in medullary thyroid carcinoma and phaeochromocytoma

**DOI:** 10.1054/bjoc.2000.1677

**Published:** 2001-03

**Authors:** L Guignat, J M Bidart, M Nocera, E Comoy, M Schlumberger, E Baudin

**Affiliations:** Department of Nuclear Medicine and Endocrine Tumors, Institut Gustave-Roussy, Villejuif, France; Department of Clinical Biology, Institut Gustave-Roussy, Villejuif, France

**Keywords:** chromogranin A, α-subunit of glycoprotein hormones, medullary thyroid carcinoma, phaeochromocytoma, neuroendocrine tumours

## Abstract

Using specific immunoradiometric assays, we evaluated the clinical usefulness of chromogranin A and the α-subunit of glycoprotein hormones in neuroendocrine tumours of neuroectodermic origin. The serum α-subunit of glycoprotein hormones was only slightly increased in 2 out of 44 medullary thyroid carcinoma or phaeochromocytoma patients with increased calcitonin or 24-hour urinary metanephrine levels. Serum chromogranin A was increased in 12 of 45 (27%) medullary thyroid carcinoma patients with an elevated calcitonin level and in 4 of 16 medullary thyroid carcinoma patients (25%) with an undetectable calcitonin level, in 5 of 7 phaeochromocytoma patients with increased urinary catecholamine and metabolite excretion, and in 2 of 3 patients with a non-functioning phaeochromocytoma. During follow-up, the course of chromogranin A was found to parallel that of tumour burden and/or 24-hour urinary metanephrine in 5 phaeochromocytoma patients. We conclude that chromogranin A measurement is not recommended for the diagnosis of medullary thyroid carcinoma patients. It may be useful in patients with functioning and non-functioning phaeochromocytomas as a follow-up marker. In neuroendocrine tumour patients with elevated calcitonin secretion, the serum α-subunit of glycoprotein hormone measurement may help differentiate medullary thyroid carcinoma or phaeochromocytoma patients from other endodermal-derived neuroendocrine tumour patients in whom it is frequently elevated. © 2001 Cancer Research Campaign http://www.bjcancer.com


*British Journal of Cancer* (2001) **84**(6), 808–812
© 2001 Cancer Research Campaign
doi: 10.1054/ bjoc.2000.1677, available online at http://www.idealibrary.com on IDEAL®

### Chromogranin A and the α-subunit of glycoprotein hormones in medullary thyroid carcinoma and phaeochromocytoma

**L Guignat[1], JM Bidart[2], M Nocera[1], E Comoy[2], M Schlumberger[1] and E Baudin[1]**

[1]Department of Nuclear Medicine and Endocrine Tumors, Institut Gustave-Roussy, Villejuif, France; [2]Department of Clinical Biology, Institut Gustave-Roussy, Villejuif, France

**Summary** Using specific immunoradiometric assays, we evaluated the clinical usefulness of chromogranin A and the α-subunit of glycoprotein hormones in neuroendocrine tumours of neuroectodermic origin. The serum α-subunit of glycoprotein hormones was only slightly increased in 2 out of 44 medullary thyroid carcinoma or phaeochromocytoma patients with increased calcitonin or 24-hour urinary metanephrine levels. Serum chromogranin A was increased in 12 of 45 (27%) medullary thyroid carcinoma patients with an elevated calcitonin level and in 4 of 16 medullary thyroid carcinoma patients (25%) with an undetectable calcitonin level, in 5 of 7 phaeochromocytoma patients with increased urinary catecholamine and metabolite excretion, and in 2 of 3 patients with a non-functioning phaeochromocytoma. During follow-up, the course of chromogranin A was found to parallel that of tumour burden and/or 24-hour urinary metanephrine in 5 phaeochromocytoma patients. We conclude that chromogranin A measurement is not recommended for the diagnosis of medullary thyroid carcinoma patients. It may be useful in patients with functioning and non-functioning phaeochromocytomas as a follow-up marker. In neuroendocrine tumour patients with elevated calcitonin secretion, the serum α-subunit of glycoprotein hormone measurement may help differentiate medullary thyroid carcinoma or phaeochromocytoma patients from other endodermal-derived neuroendocrine tumour patients in whom it is frequently elevated. © 2001 Cancer Research Campaign http://www.bjcancer.com

**Keywords**: chromogranin A; α-subunit of glycoprotein hormones; medullary thyroid carcinoma; phaeochromocytoma; neuroendocrine tumours

Hormone secretion, a main feature of neuroendocrine tumours (NET), has multiple consequences on the work-up aimed at establishing the diagnosis and the prognosis of these tumours. Furthermore, NET may be associated as part of a hereditary syndrome. A wide spectrum of so-called ‘eutopic or ectopic’ hormone secretions of varying sensitivity and specificity may be considered according to the NET primary under examination. In the cases of medullary thyroid carcinoma (MTC) and phaeochromocytoma, serum calcitonin (CT) and 24-h urinary metanephrines are respectively highly sensitive biological markers of these tumors (Ghillani et al, 1989; Héron et al, 1996) but not specific for any given NET primary (Baudin et al, 1999; Ciofu et al, 1999; Leboulleux et al, 1999). Indeed, secretion of CT and even 24-h urinary metanephrines has also been reported in endodermic-derived gastro-enteropancreatic (GEP) NET (Baudin et al, 1999; Ciofu et al, 1999). The biological characterization of NET with other hormonal markers may help determine the anatomic site of each primary. We report our experience with 2 biological NET markers, namely chromogranin A (CgA) and the α-subunit of glycoprotein hormones (GPα).

CgA detection is a major step in the diagnosis of NET (Wiedenmann and Huthner, 1989; Deftos, 1991; Öberg, 1996; Nobels et al, 1997; Ferrari et al, 1998). Elevated serum CgA levels have been demonstrated in various NET primaries with varying sensitivity (Baudin et al, 1998; Ferrari et al, 1998). Few studies have explored CgA sensitivity in MTC and phaeochromocytoma which varies between 28–100% and 76–100% respectively. Only one study has specified the relationship between CgA and CT levels in MTC patients using an in-house method for CT measurement (Blind et al, 1992) and 24-h urinary metanephrine excretion in phaeochromocytoma patients, analysed by high-performance liquid chromatography (HPLC), has seldom been considered a reference measurement technique (Héron et al, 1996). In addition, in phaeochromocytoma patients, data on the follow-up of CgA measurements are scarce.

Secretion of GPα has been reported in gastroenteropancreatic-derived NET (GEP NET), including midgut-derived NET, (Norheim et al, 1987; Öberg, 1996; Nobels et al, 1997) but also in patients with neuroectodermic-derived NET including patients with MTC and phaeochromocytoma (Nobels et al, 1997). In contrast, in a previous study investigating 130 gastro-enteropancreatic NET of various origins, we found an elevated GPα level exclusively in foregut-derived NET (Baudin et al, 1998). Moreover, in a subsequent preliminary study, we were unable to demonstrate GPα secretion in MTC patients (Leboulleux et al, 1999). We therefore put forward the hypothesis that GPα secretion might be a specific biological marker of foregut-derived NET in patients with neuroendocrine tumours. The sensitivity of GPα in neuroectodermic-derived NET had to be assessed using highly specific immunoradiometric assays (IRMA) to test this hypothesis.

We therefore conducted the present study to investigate (i) the sensitivity of GPα in patients with neuroectodermic-derived NET,

*Received 31 May 2000*
*Revised 20 December 2000*
*Accepted 21 December 2000*

*Correspondence to:* E Baudin

(MTC and phaeochromocytoma); (ii) CgA sensitivity in these tumours and its relationship with calcitonin and with 24-h urinary metanephrine measurements in MTC patients and in phaeochromocytoma patients respectively, using referenced assays and (iii) the potential interest of CgA in the follow-up of phaeochromocytoma patients.

### PATIENTS AND METHODS

### Patients

61 patients with MTC, and 17 patients with phaeochromocytoma (13 eutopic, 4 ectopic) were studied consecutively. There were 37 females and 41 males, with a mean age at diagnosis of 40 ± 12 years (mean ± SEM, range: 2–78 years). Among MTC patients, 20 patients had a familial form of the disease (either multiple endocrine neoplasia (MEN) type 2A or MEN type 2B, or familial MTC), and 41 had a sporadic MTC. All patients with MTC had undergone a thyroidectomy, and 25 patients had been resubmitted to surgery for neck or mediastinum recurrences. 8 patients had also been treated with external beam radiotherapy, and 8 with chemotherapy (Schlumberger et al, 1995). At the time of the present study, 16 patients had an undetectable plasma calcitonin (CT) level. 45 patients had an elevated CT level and were considered as having persistent disease. These 45 patients underwent the following examinations: neck and liver ultrasound (US), computed tomography (CT scan) of the thorax and abdomen and a bone scintigraphy. Distant metastases were discovered in 16 patients.

Among phaeochromocytoma patients, 5 had a familial form of the disease (either MEN 2A, Von Hippel-Lindau disease, or neurofibromatosis type 1), and 12 had a sporadic phaeochromocytoma. Ectopic phaeochromocytoma was diagnosed in 4 patients, defined as neuroendocrine tumours arising from the extra-adrenal paraganglion system. When the differential diagnosis between ectopic phaeochromocytoma and GEP NET was dubious, immunohistochemistry using epithelial markers that are positive in GEP NET but negative in phaeochromocytoma was used. All patients with phaeochromocytoma had undergone surgical removal of the tumour, and 6 had had further surgery for local or distant recurrences. 5 patients were treated with 131I-metaiodobenzyl guanidine (MIBG) and 5 with chemotherapy (Averbuch et al, 1988). The following examinations were performed in patients with persistent or recurrent disease: abdominal ultrasound, abdominal or thoracic CT scan or magnetic resonance imaging (MRI), as well as MIBG scintigraphy. Somatostatin receptor scintigraphy was performed in patients with an ectopic or a malignant phaeochromocytoma. At the time of our study, 7 patients were apparently free of disease (with normal catecholamines and metabolites and no morphological evidence of tumour) and 10 patients had persistent or recurrent disease including 7 patients with high urinary catecholamine and metabolite excretion, and 3 patients with a locoregional ($n = 1$) or a metastatic recurrence ($n = 2$) of a nonfunctioning ectopic phaeochromocytoma.

In 5 phaeochromocytoma patients with elevated CgA, serial measurements were compared to the results of morphological investigations (follow-up: 14 ± 1 months). Results were also compared to urinary metanephrine plus normetanephrine excretion in 3 of these 5 patients.

No patient had known concomitant MTC and phaeochromocytoma at the time of the study, as demonstrated by a normal CT level in phaeochromocytoma patients and normal 24-h urinary metanephrine excretion in MTC patients. Patients with renal insufficiency (serum creatinine > 125 μmol$^{-1}$ 1) were excluded.

### Methods

All samples were collected after overnight fasting and measured at the same time. Serum CgA was measured using the CgA-Riact kit (Cis Bio International, Gif-sur-Yvette, France, normal < 100 μg l$^{-1}$), a two-site immunoradiometric assay (IRMA) based on monoclonal antibodies that bind to 2 distinct epitopes within the central domain of CgA (Degorce et al, 1999). Serum GPα (normal in men and premenopausal women < 1 μg l$^{-1}$; normal in post-menopausal women < 3 μg l$^{-1}$) was measured using a specific IRMA, as previously described (Ozturk et al, 1987). CT was measured using the ELSA-hCT kit (Cis Bio International; normal < 10 ng l$^{-1}$). CEA was measured using the Enzymum-test CEA kit (Boehringer, Mannheim, Germany; normal < 7 μg l$^{-1}$). 24-h urinary catecholamine and metabolite excretion was measured using HPLC with a reverse phase column and amperometric detection, after specific extraction, as previously described (Ciofu et al, 1999). Results were indexed on 24-h urinary creatinine (metanephrine, normal < 400 nmol mmol$^{-1}$ creatinine; normetanephrine, normal < 500 nmol mmol$^{-1}$ creatinine) (Héron et al, 1996). Normal values, as defined by previous studies, were used to classify the results of these markers, as well as the 160 μg l$^{-1}$ cut-off value for CgA which is associated with a 95% specificity rate (Baudin et al, 1998). The study was performed after obtaining the informed consent of each patient.

### RESULTS

Results are summarized in Figures 1–3.

### Serum CgA levels in MTC patients (Figure 1)

Among 61 MTC patients, the serum CgA level was normal in 45 patients. It was increased in 16 patients (26%), including 12 (27%) of the 45 MTC patients with abnormal CT levels and 4 (25%) of the 16 disease-free patients with normal CT levels. Only, 2 of these 4 patients had a significantly increased CgA level (>160 μg l$^{-1}$). None of these 4 patients had a history of hypertension or

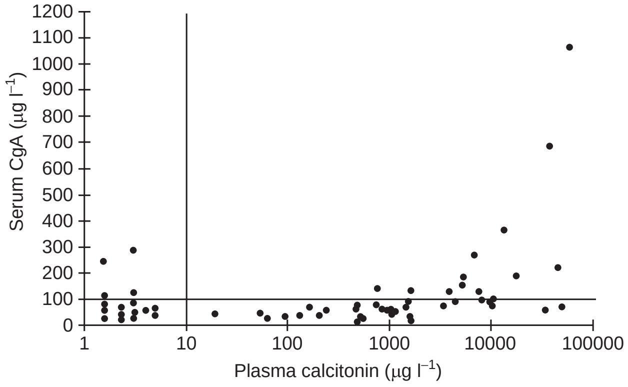

**Figure 1** Serum CgA concentrations in 61 patients with medullary thyroid carcinoma. Individual levels are represented by dots. The dash lines represent the upper limit of the normal range (CT < 10 ng l$^{-1}$, CgA < 100 μg l$^{-1}$). CT values are plotted logarithmically epigastralgy and all had a normal ionized calcaemia and PTH 1-84 levels. 12 patients had both increased CgA and CT levels: all but 1 patient had a CT level above 1000 ng $l^{-1}$ (CgA mean, 304 µg $l^{-1}$; median 185 µg $l^{-1}$; range, 126–1065 µg $l^{-1}$).

### Serum CgA levels in patients with phaeochromocytoma (Figure 2)

The CgA level was normal in all the 7 disease-free patients. Of the 7 patients with elevated urinary catecholamine and metabolite excretion, 5 (71%) had an increased CgA level, in all cases above 160 µg $l^{-1}$ (CgA mean 512 µg $l^{-1}$; median 370 µg $l^{-1}$; range, 187–1125 µg $l^{-1}$). In the 3 patients with an ectopic phaeochromocytoma and normal urinary catecholamine and metabolite excretion but with morphological evidence of recurrent disease, 2 (67%) had an elevated CgA level (> 160 µg $l^{-1}$), respectively at 386 and 1289 µg $l^{-1}$.

### CgA in the follow-up of phaeochromocytomas (Figure 3)

In the 5 phaeochromocytoma patients exhibiting elevated CgA, follow-up CgA measurements were compared to the results of morphological investigations which demonstrated one objective response in a patient who had received MIBG radionucleide therapy and progressive disease in the 4 other patients. In all cases, follow-up CgA measurements were correlated with the tumour burden. In 3 of these 5 patients, the course of follow-up CgA values was compared to the 24-h urinary metanephrine levels. A close relationship between both secretions was found in all patients, as illustrated in Figure 3.

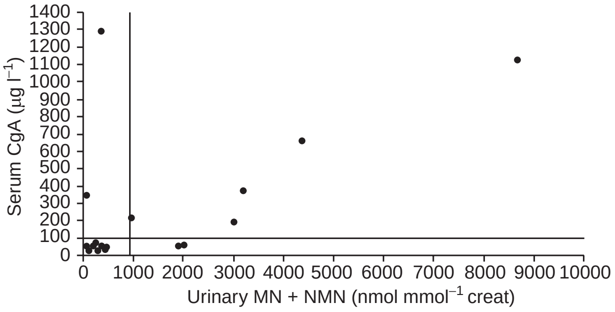

**Figure 2** Serum CgA concentrations in 17 patients with phaeochromocytoma. Individual levels are represented by dots. The dash lines represent the upper limit of the normal range (urinary MN + NMN: urinary metanephrine plus normetanephrine < 900 nmol $mmol^{-1}$ creat, CgA < 100 µg $l^{-1}$).

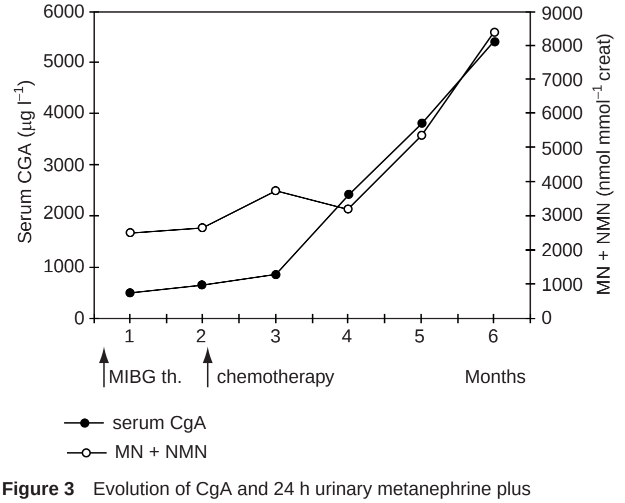

**Figure 3** Evolution of CgA and 24 h urinary metanephrine plus normetanephrine excretion in a 54-year-old man with progressive lung and liver metastases of a phaeochromocytoma

### GPα levels in patients with MTC or phaeochromocytoma

Among the 44 patients with either MTC and increased CT (*n* = 35) or recurrent phaeochromocytoma (*n* = 9), GPα was slightly elevated in only 2 women: one with a history of  µg $l^{-1}$) and one, a 38-year-old, who had a slightly increased GPα level (0.55 µg $l^{-1}$).

### DISCUSSION

In our study, serum CgA was elevated in 27% of MTC patients with hypercalcitoninaemia, and in only 56% of metastatic MTC with markedly elevated CT (> 10 000 ng $l^{-1}$). Given its low sensitivity in MTC patients, CgA does not appear to be capable of competing with CT for the diagnosis of MTC. This CgA sensitivity was in the lower range of values described in previous studies (O'Connor and Deftos, 1986; Blind et al, 1992; Nobels et al, 1997). Such discrepancies may partly be explained by different biological assays used to measure CgA. We used a 2-site 'sandwich' IRMA based on monoclonal antibody recognition of the central unprocessed domain of CgA. The results obtained with this assay may be interpreted differently from those obtained with an radioimmunologic assay based on polyclonal antibody recognition of the entire CgA molecule as well as derived peptides (Degorce et al, 1999). It is noteworthy that 2 of the 23 MTC or phaeochromocytoma patients, considered free of disease, had an elevated CgA level exceeding the 160 µg $l^{-1}$ cut-off value. The specificity of CgA in this study is therefore in the same range as that previously reported using the same immunoassay (Baudin et al, 1998). None of the classic false positive CgA results were found in these patients (O'Connor et al, 1989; Takiyyudin et al, 1990; Cryer et al, 1991; Deftos, 1991; Takiyyudin et al, 1991; Nobels et al, 1997; Sanduleanu et al, 1999). Above all associated NET as part of multiple endocrine neoplasia were excluded. As suggested by one previous study, a significant increase was found in CgA exclusively in MTC patients with advanced stage disease (Blind et al, 1992). Our study specifies the relationship between CgA and CT secretion using a sensitive commercial kit which may help physicians interpret CgA levels in these patients. All but 1 patient with a CT level below 1000 ng $l^{-1}$ were found to have a normal CgA value and only 2 MTC patients were found to have a CgA level above 400 µg $l^{-1}$. We strongly recommend therefore, that MTC patients with a significantly elevated CgA level should undergo systematic screening for an associated phaeochromocytoma as part of a multiple endocrine syndrome, type 2. Indeed, serum CgA was elevated in 70% (7/10) of patients with either a functioning or a metastatic non-functioning phaeochromocytoma. These results confirm the impact of the NET origin on CgA sensitivity. The adrenal medulla is a well-known major tissue source of CgA (Takiyyuddin et al, 1990). Previous studies reported a 76–100% sensitivity of CgA in phaeochromocytoma patients with catecholamine secretion (O'Connor and Deftos, 1986; Boosma et al,

 *© 2001 Cancer Research Campaign*

1995; Kimura et al, 1997; Nobels et al, 1997; Stidsberg and Husebye, 1997; Yanaihara et al, 1999). Apart from the various methodological techniques used to measure CgA, the characteristics of our phaeochromocytoma patient population, namely the high number of malignant and ectopic tumours, may explain the lower CgA sensitivity, found in this study. Like Hsiao et al (1991), we confirm a lower sensitivity of CgA compared to urinary metanephrine and normetanephrine, considered the most sensitive markers of phaeochromocytoma, measured with HPLC, the most reliable method of measurement (Héron et al, 1996). Previous studies have investigated the interest of CgA in phaeochromocytoma patients during the early postoperative period (Boosma et al, 1995; Stridsberg and Husebye, 1997) and one has recommended its use in this context (Stridsberg and Husebye, 1997). To our knowledge, no data have yet been published on the interest of serial CgA measurements in the follow-up of patients with malignant phaeochromocytoma. In all the 5 such patients studied, a close relationship was found between CgA levels and either the tumour burden or the course of 24-h urinary metanephrine. As serum CgA measurement can be readily obtained to monitor functioning phaeochromocytomas compared to urinary tests, we recommend that CgA be used as a follow-up marker for routine measurements in phaeochromocytoma. Of note, recent studies suggest that serum metanephrine and normetanephrine levels may have a high sensitivity in phaeochromocytoma patients (Boosma et al, 1995; Eisenhofer et al, 1999). The mechanisms of CgA secretion in patients with non-functioning phaeochromocytomas remain unclear. In these patients, CgA should also be screened by routine measurement.

No significant secretion of GPα was found in patients with either MTC or phaeochromocytoma in this study. Only 2 patients had slightly increased levels of GPα in the range of those found in postmenopausal women. These results contrast with one previous study, which found elevated GPα levels in 5 of 26 MTC, 1 of 9 phaeochromocytomas and 3 of 25 paragangliomas (Nobels et al, 1997). This discrepancy may be due to different specificities in the methods of measurement. A 2-site IRMA, using at least one monoclonal antibody directed to a specific-free GPα antigenic site was used in this study to improve the specificity of GPα measurement (Ozturk et al, 1987; Marcillac et al, 1992). Our results suggest that in NET patients with elevated CT secretion, serum GPα may be a useful marker in doubtful cases to differentiate MTC patients from foregut-derived GEP-NET patients in whom GPα is frequently elevated (Leboulleux et al, 1999).

In conclusion, we do not recommend serum CgA measurement for the diagnosis of MTC patients. CgA measurement may be useful during the follow-up of patients with phaeochromocytoma and should be screened in patients with a non-functioning phaeochromocytoma. No GPα secretion was found in MTC and phaeochromocytoma patients. In NET patients with elevated CT secretion, serum GPα measurement may help differentiate MTC or phaeochromocytoma from other GEP-NET patients.

### ACKNOWLEDGEMENTS

We are indebted to Sylvie David and Catherine Martin for secretarial assistance, the nurses of the Nuclear Medicine Department for technical assistance, and to Lorna Saint Ange for editing.

### REFERENCES

Averbuch SD, Steakley CS, Young RC, Gelmann EP, Goldstein DS, Stull R and Keiser HR (1988) Malignant phaeochromocytoma: effective treatment with a combination of cyclophosphamide, vincristine and dacarbazine. *Ann Intern Med* **109**: 267–273

Baudin E, Gigliotti A, Ducreux M, Ropers J, Comoy E, Sabourin JC, Bidart JM, Cailleux AF, Bonacci R, Ruffie P and Schlumberger M (1998) Neuron-specific enolase and chromogranin A as markers of neuroendocrine tumours. *Br J Cancer* **78**: 1102–1107

Baudin E, Bidart JM, Rougier P, Lazar V, Ruffie P, Ropers J, Ducreux M, Troalen F, Sabourin JC, Comoy E, Lasser P, DeBaere T and Schlumberger M (1999) Screening for multiple endocrine neoplasia type 1 and hormonal production in apparently sporadic neuroendocrine tumors. *J Clin Endocrinol Metab* **84**: 69–75

Blind E, Schmidt-Gayk H, Sinn HP, O'Connor DT and Raue F (1992) Chromogranin A as tumor marker in medullary thyroid carcinoma. *Thyroid* **2**: 5–10

Boosma F, Bhaggoe UM, Man in 't Veld AJ and Schalekamp MADH (1995) Sensitivity and specificity of new ELISA method for determination of chromogranin A in the diagnosis of phaeochromocytoma and neuroblastoma. *Clin Chim Acta* **239**: 57–63

Ciofu A, Baudin E, Chanson P, Cailleux AF, Comoy E, Sabourin JC, Ducreux M, Schaison G and Schlumberger M (1999) Catecholamine production in patients with gastroenteropancreatic neuroendocrine tumors. *Eur J Endocrinol* **140**: 434–437

Cryer PE, Wortsman J, Shad SD, Nowak RM and Deftos LJ (1991) Plasma chromogranin A as a marker of sympathochromaffin activity in humans. *Am J Physiol* **260**: E243–246

Deftos LJ (1991) Chromogranin A: its role in endocrine function and as an endocrine and neuroendocrine tumor marker. *Endocr Rev* **12**: 181–187

Degorce F, Goumon Y, Jacquemart L, Vidaud C, Bellanger L, Pons-Anicet D, Seguin P, Metz-Boutigue MH and Aunis D (1999) A new human chromogranin A (CgA) immunoradiometric assay involving monoclonal antibodies raised against the unprocessed central domain (145–245). *Br J Cancer* **79**: 65–71

Eisenhoffer G, Lenders JWM, Linehan M, Walther MM, Goldstein DS and Keiser HR (1999) Plasma normetanephrine and metanephrine for detecting pheochromocytoma in Von Hippel-Lindau disease and multiple endocrine neoplasia type 2. *N Engl J Med* **340**: 1872–1879

Ferrari L, Seregni E, Martinetti A, Van Graafeiland B, Nerini-Molteni S, Botti C, Artale S, Cresta S and Bombardieri E (1998) Chromogranin A measurement in neuroendocrine tumors. *Int J Biol Markers* **13**: 3–9

Ghillani PP, Motte P, Troalen F, Jullienne A, Gardet P, Le Chevallier T, Rougier P, Schlumberger M, Bohuon C and Bellet D (1989) Identification and measurement of calcitonin precursors in serum of patients with malignant diseases. *Cancer Res* **49**: 6845–6851

Héron E, Chatellier G, Billaud E, Foos E and Plouin PF (1996) The urinary metanephrine-to-creatinine ratio for the diagnosis of phaeochromocytoma. *Ann Intern Med* **125**: 300–303

Hsiao RJ, Parmer RJ, Takiyyuddin MA and O'Connor DT (1991) Chromogranin A storage and secretion: sensitivity and specificity for the diagnosis of phaeochromocytoma. *Medicine* **70**: 33–45

Kimura N, Miura W, Noshiro T, Mizunashi K, Hanew K, Shimizu K, Watanabe T, Shibukawa S, Sohn HE, Abe K, Miura Y and Nagura H (1997) Plasma chromogranin A in phaeochromocytoma, primary hyperparathyroidism and pituitary adenoma in comparison with catecholamine, parathyroid hormone and pituitary hormones. *Endocr J* **44**: 319–327

Leboulleux S, Baudin E, Young J, Caillou B, Lazar V, Pellegriti G, Ducreux M, Schaison G and Schlumberger M (1999) Gastroenteropancreatic neuroendocrine tumor metastases to the thyroid gland: differential diagnosis with medullary thyroid carcinoma. *Eur J Endocrinol* **140**: 187–191

Marcillac I, Troalen F, Bidart JM, Ghillani P, Ribrag V, Escudier B, Malassagne B, Droz JP, Lhomme C, Rougier P, Duvillard P, Prade M, Luguagne PM, Richard F, Poynard T, Bohuon C, Wands J and Bellet D (1992) Free human chorionic gonadotropin beta subunit in gonadal and nongonadal neoplasms. *Cancer Res* **52**: 3901–3907

Nobels FRE, Kwekkeboom DJ, Coopmans W, Schoenmakers HH, Lindemans J, De Herder WW, Krenning EP, Bouillon R and Lamberts SWJ (1997) Chromogranin A as serum marker for neuroendocrine neoplasia: comparison with neuron-specific enolase and the α-subunit of glycoprotein hormones. *J Clin Endocrinol Metabt* **82**: 2622–2628

Norheim I, Oberg K, Theodorsson-Norheim E, Lindgren PG, Lundqvist G, Magnusson A, Wide L and Wilander E (1987) Malignant carcinoid tumors. An analysis of 103 patients with regard to tumor localization, hormone production, and survival. *Ann Surg* **206**: 115–125

© 2001 Cancer Research Campaign 

Öberg K. (1996) The ultimate biochemical diagnosis of gastro-enteropancreatic tumours. *Digestion* **57** (suppl): 45–47

O'Connor DT and Deftos LJ (1986) Secretion of chromogranin A by peptide-producing endocrine neoplasms. *N Engl J Med* **314**: 1145–1151

O'Connor DT, Pandian MR, Carlton E, Cervenka JH and Hsiao RJ (1989) Rapid radioimmunoassay of circulating chromogranin A: in vitro stability, exploration of the neuroendocrine character of neoplasia, and assessment of the effects of organ failure. *Clin Chem* **35**: 1631–1637

Ozturk M, Bellet D, Manil L, Hennen G, Frydman R and Wands J (1987) Physiological studies of human chorionic gonadotropin (hCG), hCGα, and hCGβ as measured by specific monoclonal immunoradiometric assays. *Endocrinology* **120**: 549–558

Sanduleanu S, Stridsberg M, Jonkers D, Hameeteman W, Biemond I, Lundqvist G, Lamers C and Stockbrugger RW (1999) Serum gastrin and chromogranin A during medium- and long-term acid suppressive therapy: a case-control study. *Aliment Pharmacol Ther* **13**: 145–153

Schlumberger M, Abdelmoumene N, Delisle MJ, Couette JE and the Groupe d'Etude des tumeurs a Calcitonine (GETC) (1995) Treatment of advanced medullary thyroid cancer with an alternating combination of 5 FU-streptozocin and 5 FU-dacarbazine. *Br J Cancer* **71**: 363–365

Stidsberg M and Husebye ES (1997) Chromogranin A and chromogranin B are sensitive circulating markers for phaeochromocytoma. *Eur J Endocrinol* **136**: 67–73

Takiyyuddin MA, Cervenka JH, Pandian MR, Stuenkel CA, Neumann HP and O'Connor DT (1990) Neuroendocrine sources of chromogranin A in normal man: clues from selective stimulation of endocrine glands. *J Clin Endocrinol Metab* **71**: 360–369

Takiyyuddin MA, Baron AD, Cervenka JH, Barbosa JA, Neumann HP, Parmer RJ, Sullivan PA and O'Connor DT (1991) Suppression of chromogranin A release from neuroendocrine sources in man: pharmacological studies. *J Clin Endocrinol Metab* **72**: 616–622

Wiedenmann B and Huttner WB (1989) Synaptophysin and chromogranins / secretogranins – widespread constituents of distinct types of neuroendocrine vesicles and new tools in tumor diagnosis? *Virchows Archiv B Cell Pathol* **58**: 95–121

Yanaihara H, Hata M, Nishikawa Y, Hoshino M, Yanaihara N and Murai M (1999) Application of region-specific immunoassay for human chromogranin A: substantial clue for detection and measurement of chromogranin A in human plasma. *Regul Pept* **80**: 83–90

© 2001 Cancer Research Campaign
